# Association of Nd:YAG laser and calcium-phosphate desensitizing pastes on dentin permeability and tubule occlusion

**DOI:** 10.1590/1678-7757-2020-0736

**Published:** 2021-03-31

**Authors:** Vinícius MAXIMIANO, Alana Cristina MACHADO, Raquel Marianna LOPES, Fernanda Ellen Mercatelli RABELO, Stephanie Assimakopoulos GARÓFALO, Denise Maria ZEZELL, Ana Cecilia Corrêa ARANHA, Taís SCARAMUCCI

**Affiliations:** 1 Universidade de São Paulo Faculdade de Odontologia Departamento de Dentística São PauloSP Brasil Universidade de São Paulo, Faculdade de Odontologia, Departamento de Dentística, São Paulo, SP, Brasil.; 2 Universidade de São Paulo Instituto de Pesquisas Energéticas e Nucleares São PauloSP Brasil Universidade de São Paulo, Instituto de Pesquisas Energéticas e Nucleares (IPEN/CNEM), São Paulo, SP, Brasil.; 3 Universidade de São Paulo Faculdade de Odontologia Departamento de Dentística São PauloSP Brasil Universidade de São Paulo, Faculdade de Odontologia, Departamento de Dentística, Laboratório Especial de Laser em Odontologia (LELO), São Paulo, SP, Brasil.

**Keywords:** Dentin hypersensitivity, Laser, Desensitizing agents

## Abstract

**Objective:**

To evaluate the efficacy of Nd:YAG laser associated with calcium-phosphate desensitizing pastes on dentin permeability and tubule occlusion after erosive/abrasive challenges.

**Methodology:**

Dentin specimens were exposed to 17% ethylene diamine tetra-acetic acid (EDTA) solution for 5 min and randomly allocated into five groups: G1, control (no treatment); G2, Nd:YAG laser (1 W, 10 Hz, 100 mJ, 85 J/cm2); G3, Laser + TeethmateTM Desensitizer; G4, Laser + Desensibilize Nano P; and G5, Laser+Nupro^®^. Specimens underwent a 5-day erosion-abrasion cycling. Hydraulic conductance was measured post-EDTA, post-treatment, and post-cycling. Post-treatment and post-cycling permeability (%Lp) was calculated based on post-EDTA measurements, considered 100%. Open dentin tubules (ODT) were calculated at the abovementioned experimental moments using scanning electron microscopy and ImageJ software (n=10). Data were analyzed using two-way repeated measures ANOVA and Tukey’s test (α=0.05).

**Results:**

G1 presented the highest %Lp post-treatment of all groups (p<0.05), without significantly differences among them. At post-cycling, %Lp significantly decreased in G1, showed no significant differences from post-treatment in G3 and G4, and increased in G2 and G5, without significant differences from G1 (p>0.05). We found no significant differences in ODT among groups (p>0.05) post-EDTA. At post-treatment, treated groups did not differ from each other, but presented lower ODT than G1 (p<0.001). As for post-cycling, we verified no differences among groups (p>0.05), although ODT was significantly lower for all groups when compared to post-EDTA values (p<0.001).

**Conclusion:**

All treatments effectively reduced dentin permeability and promoted tubule occlusion after application.

**Combining Nd:**

YAG laser with calcium-phosphate pastes did not improve the laser effect. After erosive-abrasive challenges, treatments presented no differences when compared to the control.

## Introduction

Dentin hypersensitivity (DH) is a common painful condition defined as a short and acute pain resulting from exposed dentin in response to external stimuli, such as thermal, osmotic, tactile, chemical, or evaporative, and unrelated to any other dental disease or defect.^1^ DH prevalence may vary widely among studies, but can be as high as 89.1%.^2^ Studies have demonstrated that sensitive teeth have more exposed dentin tubules and with larger diameter than those of non-sensitive teeth, so that the patency of dentine tubules is an important and necessary condition for DH to occur.^3^ Formulated by Brännström and Aström^4^ in 1972, the hydrodynamic theory postulates that nociceptors located at the pulp-dentin interface can be activated by stimuli-induced changes in dentin fluid flow. Based on this theory, occluding dentinal tubules and reducing dentin permeability are deemed a rational approach to avoid or disable triggers that cause pain.

Many are the desensitizers available for treating DH, each with a different mechanism of action and effectiveness. However, no consensus has been reached about the best treatment for this condition. Fluoride, oxalates, strontium, calcium/phosphate formulations, and lasers are some of the agents used to manage DH.^5^ For being able to occlude dentinal tubules by mineral deposition, calcium and phosphate-based products are promising options.^6^ Although efficiently used to promote immediate relief,^7^ some of these agents lack the necessary ability to resist erosive-abrasive challenges.^8^ Associating treatments has shown to reach superior results in tubule occlusion, such as the use of high-power lasers along with desensitizing agents.^9^ However, their effectiveness has not always been shown to be greater in the long-term when compared to a single treatment.^10^

Among high-power lasers, Nd:YAG has been widely used to treat DH.^11^ Nd:YAG laser features thermal effects by melting and re-solidifying dentin surface, obliterating or narrowing dentinal tubules and forming a glazed surface.^12^ When compared to other lasers,^13^ Nd:YAG presents superior effects on reducing pain and obliterating tubules by promoting homogeneous melting^12^. This laser was considered safe, resulting in no pulp damage or tissue cracks and craters.^14^ Manual dentin irradiation may result in areas without morphological changes and with open tubules, once the irradiation may not cover the whole surface equally.^15^ Considering that irradiation is always performed manually in the daily practice, applying another occluding agent post-irradiation may help to improve tubule occlusion, reducing dentin permeability.

This *in vitro* study aimed to evaluate the efficacy of Nd:YAG laser associated with three calcium and phosphate-based desensitizing agents – Tetracalcium phosphate and Dicalcium phosphate anhydrous (Teethmate^TM^ Desensitizer); Nano-hydroxyapatite (Desensibilize Nano P); and sodium calcium phosphosilicate (Nupro^®^ paste – NovaMin^®^) – in occluding dentinal tubules and reducing dentin permeability immediately after application and after erosive-abrasive challenges. Our null hypotheses were: 1. Groups would present no differences in dentin permeability and tubule occlusion after treatment; and 2. Groups would present no differences in dentin permeability and tubule occlusion after erosive-abrasive cycling.

## Methodology

**Figure 1:**
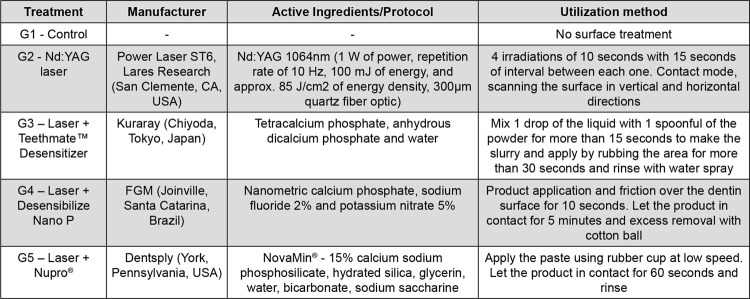
In-office desensitizing treatments: description of manufacturers, active ingredients and utilization method

Based on a completely randomized design, this study included two experimental factors: desensitizing treatment and experimental moment. The desensitizing treatment factor comprised five groups (Figure 1): G1, control (no treatment); G2, Nd:YAG laser (Power Laser, Lares Research, San Clemente, CA, USA – process FAPESP 07/55497-0); G3, Nd:YAG + Teethmate™ Desensitizer (Kuraray, Chiyoda, Tokyo, Japan); G4, Nd:YAG + Desensibilize Nano P (FGM, Joinville, Santa Catarina, Brazil); and G5, Nd:YAG+Nupro^®^ prophy paste (Dentsply, York, Pennsylvania, USA). The hydraulic conductance of each group was analyzed at two experimental moments: post-treatment and post-cycling. Environmental scanning electron microscopy (ESEM) (Hitachi Analytical Table Top Microscope TM3000, Hitachi, Tokyo, Japan) was performed at three moments: post-EDTA (baseline), post-treatment, and post-cycling. Each group contained 10 human dentin specimens (n=50 for hydraulic conductance and n=50 for ESEM). Hydraulic conductance analysis measured permeability (% Lp, determined based on the post-EDTA permeability), and ESEM qualitatively measured the number of open dentin tubules (ODT) by visual analysis and quantitatively by the ImageJ software.

### Specimen Preparation - Dentin permeability and ESEM

This study was approved by the local Ethics Committee on Research with Humans Beings (Process #2.340.539). After being visualized under a stereomicroscope (Meiji 2000 with magnification of 10x), 70 sound human molars were selected and cleaned with scalers to remove gingival tissue and residual calculus. Then, crowns and roots were separated – crowns for the dentin permeability analysis and roots for the ESEM evaluation. Two parallel sections were made approximately 1.5 mm apart in the middle area of the crowns, perpendicularly to the long axis of the tooth. Dentine discs were sectioned, avoiding pulp horns and the occlusal enamel. At root’s middle region, dentin slabs (4 mmx4 mmx2 mm) were obtained, by performing two sections perpendicular to the long axis and two parallel to the long axis, using a precision cutting machine (Isomet 1000, Buehler Ltd, Lake Buff, Illinois, USA). Dentin specimens with fractures, restorative materials, carious lesions, or any other abnormality were discarded. Dentin surfaces were then polished in a polishing machine (Buehler Ltd, Lake Buff, Illinois, USA) using a 600-grit abrasive disc at 1N pressure and under constant water cooling until all specimens were 1 mm thick. A digital caliper was used (Mitutoyo, Tokyo, Japan) to check specimens’ thickness. At the end of polishing procedure, the specimens were sonicated (Ultrassonic Bath, Cristófoli, Campo Mourão, Paraná, Brazil) for 3 minutes in distilled water to remove debris.

### Dentin hypersensitivity simulation

To open dentin tubules and simulate a hypersensitive condition, all specimens were immersed in 17% ethylene diamine tetra-acetic acid (EDTA) solution (pH 7.4) for 5 min,^7^ rinsed with distilled water, and stored in relative humidity at 4°C. Dentin permeability and tubule counting obtained after this procedure were considered as baseline values. Then, the specimens were randomly allocated into different groups (n = 10 for dentin permeability and n=10 for ESEM evaluation).

### Application of the treatments

Specimens were taken off the relative humidity environment and gently wiped with a soft absorbent paper to remove excess water from the surface. Then, each treatment was administered on specimens polished occlusal surface following laser protocols that showed good results in the literature^7,11^ and manufacturer’s instructions for desensitizing agents, as shown in Figure 1. Specimens from control group were gently wiped, as previously described, but received no subsequent treatment. Until post-treatment analyses, treated specimens were stored in a humid environment at 4°C, achieved by wrapping specimens in soft tissues moisturized with distilled water and enclosing them in containers. Groups with treatments association underwent Nd:YAG laser irradiation before the application of the desensitizing agent, according to the protocol described in Figure 1.

### Erosive-abrasive cycle

We simulated the natural intraoral environment through the 5-day erosive-abrasive cycling.^7^ First, specimens were immersed for 2 min in citric acid (0.3%, natural pH, approx. 2.6) and then remineralized for 60 min in artificial saliva (0.213 g/l CaCl_2_·2H_2_O; 0.738 g/l KH_2_PO_4_; 1.114 g/l KCl; 0.381 g/l NaCl; 12 g/l Tris buffer, pH adjusted to 7.0 with HCl)^16^ under constant agitation (35 rpm, orbital shaker, AI9000IB, BrILabs). The erosion-remineralization cycling was performed four times a day. Thirty minutes after the first and last erosion challenges, specimens were brushed for 15 s in an automatic brushing machine (45 strokes – each stroke representing a back-and-forth movement under a 2 N load; total exposure time to the slurries was 2 min). Toothbrushing was performed with standard brushes (Tek, soft bristles, Johnson & Johnson, Brazil) and conventional fluoride toothpaste (Colgate Maximum Caries Protection, Colgate Palmolive, Brazil, sodium monofluorophosphate, 1450 ppm F^-^) diluted in distilled water (1:3 ratio). After each erosive/abrasive cycle, specimens were washed with distilled water and gently wiped with absorbent paper before being immersed in artificial saliva. All experimental procedures were performed at room temperature (approx. 23°C). At the end of each cycle, specimens were kept in artificial saliva under constant agitation until the next cycling procedures. The citric acid solution was replaced after erosive challenges and the artificial saliva before a new cycle.

After five days of erosive-abrasive cycling, dentin permeability (%LP), qualitative surface evaluation, and open tubule counting (ODT) by environmental scanning electronic microscopy (ESEM) were performed.

### Dentin permeability analysis

Dentin permeability was assessed through hydraulic conductance using an equipment that simulate intrapulpal pressure (Odeme Equipamentos Médicos e Odontológicos Ltda, Luzerna, Brazil). For that, dentin discs (n=10) were positioned in the machine flow chamber with the occlusal surface facing upward. Inside the system, the water flow through the dentin disc, from the pulp-facing side to the occlusal surface, simulating intrapulpal pressure. The system was maintained under constant pressure of 10 psi during the experiment. At each analysis, water flow was measured for 3 min by the linear displacement (mm) within the microcapillary glass (100 µl) of an air bubble, replaced after each analysis. Measurements were repeated three times for each specimen, and the average was calculated. Results were converted into flow volume (µl mim^1^) and then into hydraulic conductance Lp (mim^-1^ cm^2^cmH^2^O^-1^).^17^ The measured hydraulic conductance considered the flow volume previously calculated, the area through which the water passed in the specimen dentin disc (0.058 cm^2^), and the system hydrostatic pressure (10 psi) converted into cmH_2_O (703.07 cmH_2_O). Each specimen Lp values were calculated after treatment and after cycling, and hydraulic conductance (%Lp) was calculated based on baseline values (post-EDTA, which was considered 100%). In this case, each specimen served as its own control.^18^

### Environmental Scanning Electron Microscopy (ESEM)

Specimens were qualitatively and quantitatively analyzed by ESEM (Hitachi TM3000, Hitachi, Tokyo, Japan) after EDTA, treatment, and cycling, without sample preparation. Representative micrographs at 2,000x magnification were taken at the center of each specimen. Quantitative analysis was conducted as previously described,^19^ and the number of open dentin tubules (ODT) was calculated using the ImageJ software (n=10 for each group).

### Statistical analyses

Data on %Lp and ESEM were analyzed for normal distribution using the Shapiro-Wilk test and for homoscedasticity using the Brown-Forsythe test. After verifying data normality and homoscedasticity, the experimental groups were compared with two-way repeated measures ANOVA and Tukey’s test. Significance level was set at 5%. All statistical analyses were performed with SigmaPlot 13 software (Systat Software Inc., USA).

## RESULTS

### Dentin permeability

We found no significant differences for dentin permeability among desensitizing treatments (p=0.139), but experimental moments (p<0.001) and interaction between factors (p<0.001) significantly differed.

**Figure 2:**
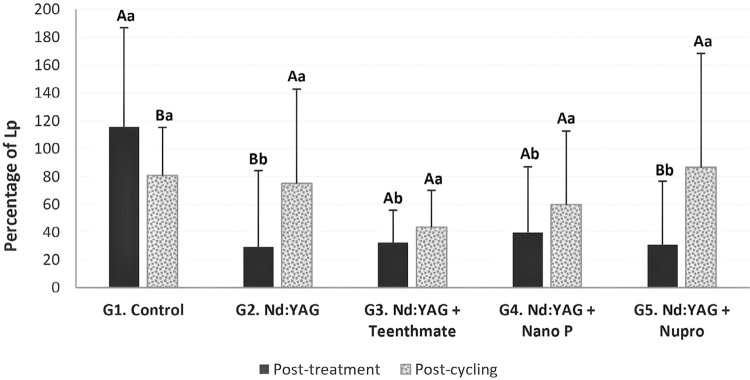
Means (standard deviations) of %Lp values for all groups at both experimental moments. Different Capital letters imply differences between experimental moments within groups (p≤0.05). Different lowercase letters denote differences among groups within moments (p≤0.05)

Figure 2 shows the mean (standard deviations) permeability (%Lp) for all groups at post-treatment and post-cycling. Regarding desensitizing treatments, all treatments presented significant lower %Lp than the control group (G1) at post-treatment (p<0.05), but without significant differences among treatments (p>0.05). At post-cycling, groups showed no significant differences (p>0.05). When comparing both experimental moments (post-treatment and post-cycling), we found only G3 and G4 to show no significant differences in %Lp (p>0.05) – post-cycling %Lp was lower in G1 and higher in G2 and G5.

### Scanning Electron Microscopy

#### Quantitative evaluation

The quantitative analysis found the number of open dentin tubules (ODT) to significantly differ among desensitizing treatments (p<0.001) and experimental moments (p<0.001). We also verified a significant difference considering the interaction between factors (p=0.007).

**Figure 3:**
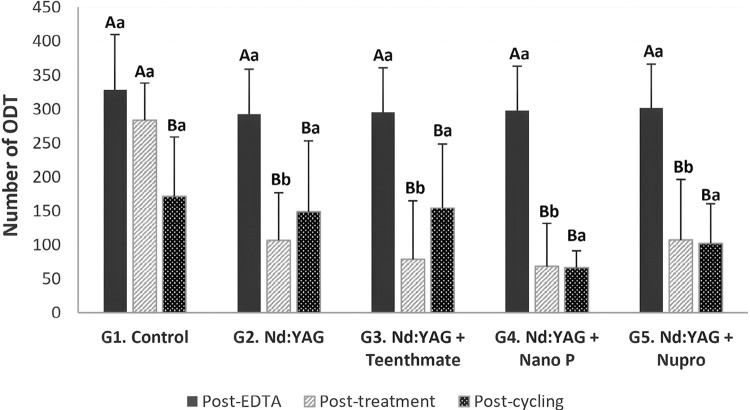
Means (standard deviations) of ODT for all groups in all experimental moments. Different Capital letters imply differences between experimental moments within groups (p≤0.05). Different lowercase letters denote differences among groups within moments (p≤0.05)

Figure 3 shows the means and standard deviations of ODT for all groups at post-EDTA (baseline), post-treatment, and post-cycling. We found no differences in ODT among groups post-EDTA (p>0.05). At post-treatment, G1 presented a significantly higher number of ODT (p<0.001) than treatment groups, but without significant differences among these (p>0.05). At post-cycling, ODT counting did not significantly differ among groups (p>0.05). By comparing experimental moments, we found all groups to show significant differences between post-EDTA and post-treatment values (p<0.001) except G1 (p>0.05), which presented significantly lower post-cycling ODT (p<0.05) when compared to post-treatment. Post-cycling ODT was not significantly different in the other treatment groups. Comparisons between post-EDTA and post-cycling showed a significant decrease in ODT counting for all groups (p ≤ 0.001).

#### Qualitative evaluation

**Figure 4:**
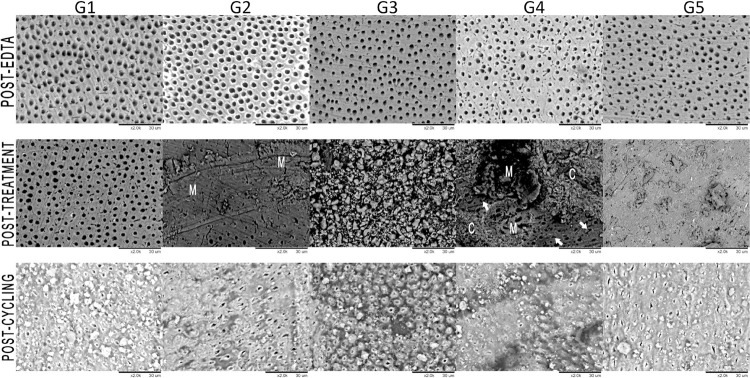
Representative micrographs of treatments for each group and evaluation moment (2000x magnification). (M) Melting; (C) Crystal-like layer. Arrows indicate crystals deposition inside dentin tubules

Figure 4 shows all representative micrographs. At post-EDTA, all groups showed the dentin surfaces without smear-layer and with a high number of ODT. Post-treatment, the control group (G1) maintained the same characteristics, presenting opened and exposed dentin tubules. G2 specimens showed dentin melting areas and an irregular surface with obliterated and narrowed tubules, although areas with opened tubules were also present. G3 surface presented the formation of a crystal-like layer, with a wide range of crystals diameters. In areas without crystal coverage, we observed opened and narrowed tubules and dentin melting areas. G4 specimens’ surfaces showed dentin melting areas (indicated by M in Figure 4) associated with small crystal-like deposits (indicated by C in Figure 4), besides areas with opened tubules, unreached by the treatment. These crystal-like deposits seemed smaller than those found in G3 and G5. Moreover, many surface areas showed a large number of visible tubules amidst the crystal layer. G5 presented the formation of a heterogeneous crystal-like layer with completely or partially occluded tubules. In areas with large crystals, opened tubules could be seen through these mineral deposits.

Post-cycling, G1 presented a smear layer-covered surface with most dentin tubules obliterated or narrowed. G2 specimens showed some surface cracks and open dentin tubules, although apparently small in diameter than those observed post-EDTA, as well as areas with smear layer and granules obliterating some tubules. All the groups with treatment associations (G3, G4, G5) presented some obliterated tubules and many areas with totally or partially opened tubules, as well as smear layer and granule deposits on the surface.

## Discussion

Considering the pain mechanism described by the hydrodynamic theory,^4^ the literature tends to agree that reducing the number of open dentin tubules may minimize pain and treat dentin hypersensitivity (DH).^20^ Our study employed a model capable of evaluating dentin permeability and tubule occlusion, thus suitable for assessing the efficacy of the treatments tested. For simulating pulpal pressure and expressing results in objective and quantitative values, hydraulic conductance is an effective method to evaluate dentin permeability. In turn, environmental scanning electron microscopy (ESEM) offers information on the dentin surface topography, allowing us to assess its morphology and the magnitude of tubular occlusion. This method requires no sample preparation for analyzing dentin permeability, so that the same specimen could be measured at different time-points. Data on dentin permeability and tubule occlusion showed similar outcomes in our study.

We assessed the efficacy of Nd:YAG laser associated with calcium phosphate-based pastes in reducing dentin permeability and promoting tubule occlusion after treatment. Our results show that all treatments achieved the desired goal, promoting lower dentin permeability and tubules occlusion when compared with post-EDTA (baseline), when dentin permeability was considered 100%. Immediately after application, all treatments showed lower ODT and %LP than the control group. These findings indicate that our first study null hypothesis was rejected. We also found no significant differences among treatments, suggesting they were equally effective. Considering that, the laser effect was not improved in association with treatments.

Evidence suggests that Nd:YAG laser changes the dentin surface, which could be relevant for treating DH.^15^ Nd:YAG irradiation increases dentin surface temperature, causing its melting and immediate re-solidification and consequently promoting tubule occlusion. Corroborating previous studies,^5,9^ our results show that the Nd:YAG laser irradiation can reduce permeability and increase tubule occlusion. This laser irradiates by scanning the entire dentin surface through an optic fiber perpendicular to it. Although we endeavored to ensure most of the dentin surface would receive proper treatment, post-treatment ESEM images showed regions without morphological changes in open tubules, probably unreached by the optic fiber. As reported by a previous study,^18^ this was the downside inherent to manual handling the laser.^18^ Based on a previous study that reported pastes isolated effect,^7^ we assumed that combining laser with the pastes would result in a complementary effect, occluding tubules that remained open after irradiation. However, that was not the outcome observed. Although all specimens treated with Nd:YAG laser still presented some opened tubules in unirradiated areas, laser treatment might have been sufficiently efficient so that no additional effect was achieved by associating treatments. We could also assume that, differently from varnishes or more fluid desensitizers, pastes consistency precluded a greater surface coverage.^19^ We set the Nd:YAG power at 100 mJ, 1 W, 10 Hz, ≈85 J/cm^2^, which, according to previous findings, has proved to be safe and efficient for dentin occlusion^10^.

Recently discovered and little tested,^21^ teethmate desensitizer (TD) is a mildly alkaline material that contains a tetracalcium phosphate (TTCP) and dicalcium phosphate anhydrous (DCPA) powder that transforms into hydroxyapatite (HA) when exposed to water or an aqueous solution, such as saliva.^21^ An interesting feature of TD is that TTCP/DCPA dissolution supplies a supersaturated calcium and phosphate ion solution, favoring HA continued precipitation within the oral environment.^22^ Calcium phosphate precipitation seems to occlude dentin tubules at 10-15 µm depth, and fluoride helps transforming DCPA into hydroxyapatite.^23^ In our study, G3 revealed a heterogeneous crystal-layer deposit on dentin surface, melted dentin areas, and nearly no open tubules apparent in post-treatment ESEM, as well as some mineral crystals within dentin tubules. TD and Nd:YAG laser association showed to effectively narrow and occlude dentin tubules after treatment, corroborating previous studies addressing TD potential (when used alone) in reducing dentin permeability.^21,24^

Nano-hydroxyapatite (N-HA) – the desensitizing agent present in Desensibilize Nano P (DNP) paste – is very similar to the dental apatite^25^ and considered one of the most biocompatible materials,^26^ so that it has been employed in bone regeneration and tooth remineralization.^26^ When applied over dentin, N-HA effectively reduces dentin permeability by promoting remineralization and dentinal tubules occlusion.^27^ In our study, Nd:YAG laser and DNP association (G4) showed a significant reduction in %Lp and ODT values after treatment, significantly lower than control. G4 micrographs exhibited areas of melting interleaved with crystal-like deposits, where dentin tubules were not apparent. However, we observed opened dentin tubules in several other areas, indicating that treatments were unable to cover the surface homogeneously. Despite that, we verified the presence of crystals within some dentin tubules, indicating that N-HA may have narrowed them (arrows in Figure 4). Dentin permeability results are compatible with ODT counting and ESEM qualitative evaluation.

Nupro^®^ prophylaxis paste (NU) contains 15% Novamin^®^ (the commercial name of calcium sodium phosphosilicate - CSP). In aqueous environment, CSP releases calcium and phosphate ions that react to form hydroxycarbonate apatite (HCA), a product chemically similar to dental HA whose deposition forms a crystal layer capable of occluding dentin tubules.^28^ At post-treatment ESEM analyses, G5 images showed most of the dentin surface to be covered by a crystal-like layer; the few tubules apparent were also partially occluded. We did not observe melting areas, suggesting that HCA paste deposition overlaid laser irradiation effects. This could be deemed as a good effect of the treatment association, as untreated areas were widely sparse. We found no differences for %Lp and ODT counting between G5 and the other treatment groups, corroborating previous studies that also tested Nd:YAG laser and CSP paste association.^5,29^

Ideally, DH treatment should provide long-term tubule occlusion and resistance to the erosive and abrasive challenges constantly present in the oral environment.^21^ Our post-cycling results showed no significant differences among all groups (including the control) for %Lp and ODT counting, indicating that our second null hypothesis was accepted. These finding may be partially explained by the control group %Lp results, significantly lower in post-cycling when compared to post-treatment. The erosive-abrasive cycling probably caused abrasive particles from the toothpaste to deposit on dentin tubules, occluding them with a smear layer and consequently reducing dentin permeability.^30^ Moreover, the fluoride present in the toothpaste formulation may have contributed to tubule occlusion, indicating that the lack of differences among groups post-cycling could be due to G1 results. Given that such effect was present in all groups, it cannot be considered a confounding factor. For ESEM evaluation, G1 showed a reduction in ODT post-cycling, and the micrographs exhibited partially or totally occluded dentin tubules, corroborating the findings for %Lp.

In G2, %Lp values increased in post-cycling when compared to post-treatment, but we verified no differences regarding ODT for these two experimental moments, and its micrographs showed narrowed dentin tubules and some crystals scattered over the surface. The sealing depth of Nd:YAG laser in dentin tubules was nearly 4 µm.^12^ The erosive-abrasive cycling model used in our study was adapted from Machado et al.^7^ (2019) and attempted to simulate a high dietary intake of acid 4 times a day, with 2 abrasive challenges, causing a dentin surface loss greater than 10µm.^31^ Thus, the erosive-abrasive cycling removed nearly all laser-treated surface, partially re-opening dentin tubules and causing %Lp values to increase as the tubules were re-exposed. Post-cycling ODT counting was not significantly different from post-treatment. This may be explained by the melting residual effect or by the deposition of toothpaste abrasives, which created a smear layer that was not firmly attached to the dentin surface. Although our study failed in showing the long-term effects of Nd:YAG laser, clinical trials have reported Nd:YAG laser irradiation to maintain its desensitizing action from 4 weeks to 6 months of follow-up^32^. This case should consider the laser effect of promoting a temporary change at the pulp sensory axon endings, resulting in analgesia.^33^

In G3, post-cycling %Lp and ODT values did not significantly differ from those recorded at post-treatment. Qualitative ESEM observation revealed partially opened tubules and an intense precipitated layer deposition narrowing tubules, as well as some smear on the dentin surface. These results indicate that the occluding effect of Nd:YAG laser associated with TD may have lasted post-cycling, although the values did not significantly differ from G1 post-cycling. Micrographs showed that tubules partial obliteration effectively maintained %Lp values even after cycling. The superficial crystal deposits visible at post-treatment were removed after the erosive-abrasive challenges, but the crystals within tubules opening resisted. Once in contact with the aqueous environment and with fluoride from the toothpaste, the residual TTCP/DCPA released calcium phosphate and caused the precipitation of fluorapatite-like crystals, narrowing dentin tubules. This continuous crystal precipitation was demonstrated in previous studies.^34^

N-HA has been proposed to act through a dual mechanism, causing tubular deposition by direct deposition and intratubular mineralization after reacting with existing intraoral ions.^35^ G4 post-cycling %Lp and ODT values did not significantly differ from those recorded at post-treatment. ESEM images showed occluded and partially opened dentin tubules, with an intense crystal-like layer deposition. Although this group results do not differ from those of G1 at this experimental moment, these findings suggest that Nd:YAG laser associated with DNP can partially resist to the erosive-abrasive cycling. N-HA ability to resist to erosive cycling has been already described in the literature.^35^ Given that occluded dentin tubules are directly related to lower dentin permeability, %Lp results corroborate ESEM observations. G3 and G4 were the only two groups to show no significant differences for %Lp and ODT post-cycling in relation to post-treatment.

As for G5, post-cycling %LP significantly increased compared to post-treatment, but without significant differences when compared to G1. Conversely, post-cycling ODT counting did no differ from post-treatment, and ESEM images showed narrowed tubules and some smear on dentin surface. These findings allow us to infer that, regardless of the partial occlusion observed on dentin tubules, this effect failed in preventing post-cycling increase in dentin permeability. We could hypothesize that, although some dentin tubules were occluded, crystals and precipitates were not strongly attached to the dentin surface, being removed by the pressurized water flow during the dentin permeability experiment. The results for Nd:YAG laser and NU paste association corroborate those reported by previous studies.^19^

Both analytical methods evaluated but a small area, which may be considered a limitation of this study given that it could lead to an underestimation or overestimation of the treatments effect.^18^ When compared to baseline results (post-EDTA), all treatments presented significantly lower %Lp and ODT values at post-cycling. All treatments were administered according with the manufacturer’s instructions to cover the complete dentin surface. However, ESEM observations showed treatments to obtain a heterogeneous distribution on the dentin surface for all groups, evincing the difficulty in treating areas with exposed dentin. The treatments used in this study may present other agents capable of acting on DH, such as the potassium nitrate from Desensibilize Nano P^36^ or the analgesic effect of Nd:YAG irradiation, which could contribute in relieving pain.^33^ As we opted by not including groups treated with pastes only in the experiment, we cannot judge whether the melting promoted by laser could have negatively influenced pastes effect. All these aspects should be considered when extrapolating this study results to the clinical scenario.

## Conclusions

The association between Nd:YAG laser and calcium and phosphate-based pastes effectively reduced dentin permeability and the number of open dentin tubules immediately after application. However, the values recorded post-treatment did not differ from the control group after 5 days of erosive-abrasive cycling.
